# The stage-regulated HASPB and SHERP proteins are essential for differentiation of the protozoan parasite *Leishmania major* in its sand fly vector, *Phlebotomus papatasi*

**DOI:** 10.1111/j.1462-5822.2010.01507.x

**Published:** 2010-12

**Authors:** Jovana Sádlová, Helen P Price, Barbara A Smith, Jan Votýpka, Petr Volf, Deborah F Smith

**Affiliations:** 1 Department of Parasitology, Faculty of Science, Charles UniversityPrague CZ 128 44, Czech Republic; 2 Centre for Immunology and Infection, Department of Biology/Hull York Medical School, University of YorkYork YO10 5DD, UK

## Abstract

The stage-regulated HASPB and SHERP proteins of *Leishmania major* are predominantly expressed in cultured metacyclic parasites that are competent for macrophage uptake and survival. The role of these proteins in parasite development in the sand fly vector has not been explored, however. Here, we confirm that expression of HASPB is detected only in vector metacyclic stages, correlating with the expression of metacyclic-specific lipophosphoglycan and providing the first definitive protein marker for this infective sand fly stage. Similarly, SHERP is expressed in vector metacyclics but is also detected at low levels in the preceding short promastigote stage. Using genetically modified parasites lacking or complemented for the LmcDNA16 locus on chromosome 23 that contains the HASP and SHERP genes, we further show that the presence of this locus is essential for parasite differentiation to the metacyclic stage in *Phlebotomus papatasi*. While wild-type and complemented parasites transform normally in late-stage infections, generating metacyclic promastigotes and colonizing the sand fly stomodeal valve, null parasites accumulate at the earlier elongated nectomonad stage of development within the abdominal and thoracic midgut of the sand fly. Complementation with HASPB or SHERP alone suggests that HASPB is the dominant effector molecule in this process.

## Introduction

Kinetoplastid parasites of the genus *Leishmania* cause a diverse spectrum of infectious diseases, the leishmaniases, in tropical and subtropical regions of the world (Murray *et al*., 2005; Chappuis *et al*., 2007; Reithinger *et al*., 2007). Mammalian-infective *Leishmania* species are divided into two subgenera, *Leishmania* (*Leishmania*) and *Leishmania* (*Viannia*), that partially differ in their developmental cycles within the female sand fly vector (reviewed in Lainson *et al*., 1987). In both subgenera, however, *Leishmania* undergo transformation from the intracellular amastigotes taken up in the sand fly blood meal to flagellated promastigotes of different morphological forms (described below, using the terminology of Walters, 1993 and Cihakova and Volf, 1997). Completion of the parasite life cycle by transmission from vector to mammalian host requires promastigote differentiation into non-replicative metacyclic parasites. These forms are inoculated when the female sand fly takes a second blood meal (Bates, 2007); the parasites enter resident dermal macrophages and transform into replicative amastigotes that can be disseminated to other tissues, often inducing immuno-inflammatory responses and persistent infection. The fate of these intracellular parasites determines disease type, which can range from cutaneous or mucocutaneous infection to diffuse cutaneous or the potentially fatal visceral leishmaniasis (Murray *et al*., 2005; Chappuis *et al*., 2007; Reithinger *et al*., 2007).

Metacyclogenesis in *Leishmania*, the end-point of parasite development in the vector, is induced *in vitro* by low pH and nutrient depletion, while reduced tetrahydrobiopterin levels may also act as a signal for parasite differentiation (Cunningham *et al*., 2001; Kumar *et al*., 2007; reviewed in Bates, 2008). Metacyclic parasites display distinctive morphological and biochemical features: they have a small cell body and relatively long flagellum, are highly motile and resistant to human complement, therebyfacilitating parasite survival in the host following transmission (Da Silva *et al*., 1989). Complement resistance is associated with presence of an extensive glycocalyx composed chiefly of a complex lipid-anchored glycoconjugate, lipophosphoglycan (LPG; Turco and Descoteaux, 1992). Stage-specific expression of LPG and the parasite surface metalloprotease, GP63, have been monitored in sand flies by immunohistochemistry (Davies *et al*., 1990; Saraiva *et al*., 1995). Importantly, it has been shown that modification of LPG during metacyclogenesis facilitates detachment of *Leishmania major* from the midgut in the sand fly species, *Phlebotomus papatasi*, and is essential for vector transmission (Sacks and Kamhawi, 2001; Kamhawi, 2006). Relatively little is known, however, of parasite factors that promote later stages of development and their role in metacyclic transmission. Here we show that the stage-specific HASP (hydrophilic acylated surface protein) and SHERP (small hydrophilic ER-associated protein) proteins of *L. major* are essential for metacyclogenesis in the vector *P. papatasi*.

HASP and SHERP genes are encoded on the same segment of chromosome 23 (originally named the LmcDNA16 locus; Flinn and Smith, 1992) in both Old and New World *Leishmania* species, while similar but divergent sequences are found at the same location in the genome of *L.* (*Viannia*) *braziliensis* (D. Depledge, unpublished). The *Leishmania*-specific HASPs, characterized mainly by work on HASPB, are expressed on the plasma membrane of infective parasite stages only (metacyclics and amastigotes) and show both inter- and intra-specific variation, principally in their characteristic repetitive amino acid domains (Flinn *et al*., 1994; Rangarajan *et al*., 1995; McKean *et al*., 1997a;Alce *et al*., 1999; Denny *et al*., 2000). The smallest HASPs, the HASPAs (McKean *et al*., 1997b), lack these repeats, which are the only HASP domains showing some similarity to other proteins: specifically to the peptidoglycan- and immunoglobulin-binding domains of several bacterial surface proteins (Flinn *et al*., 1994). The HASPs are dually acylated by the N-terminal addition of myristate and palmitate, co- and post-translational modifications that are essential for plasma membrane trafficking (Denny *et al*., 2000). HASP function has been investigated in mutant parasites generated by targeted deletion of the whole LmcDNA16 locus (McKean *et al*., 2001). *In vitro*, these null parasites can undergo metacyclogenesis, are taken up by macrophages and survive in numbers comparable to wild type; *in vivo* they cause more rapid infection than wild-type parasites in susceptible BALB/c mice. In contrast, null parasites complemented by re-expression of the LmcDNA16 locus from an episome (that causes constitutive overexpression) are completely avirulent, probably due to pleiotrophic effects (McKean *et al*., 2001).

The LmcDNA16 null parasites described above are also deleted for the SHERP genes, found in close proximity within the HASP locus in all *Leishmania* species examined to date. SHERP is expressed in metacyclic parasites in culture, being the only well-validated protein marker exclusive to this stage (and not expressed in amastigotes; Knuepfer *et al*., 2001). The SHERP open reading frame is expressed as a 6.2 kDa peripheral membrane protein that localizes, in wild-type metacyclics, to the cytosolic face of the ER and the outer mitochondrial membrane. Recent structural analysis suggests that SHERP is induced to fold by interaction with membrane lipids (B. Moore, unpublished) but the function of this unusual small protein is otherwise unknown.

Given the specific and high-level expression of HASP and SHERP products in metacyclics, and the critical role of these parasite stages in successful parasite transmission, we are using null and complemented mutant lines to investigate HASPB and SHERP function in the sand fly. Here, we confirm that stage-specific expression of each protein, as observed in culture, is also found in the vector although the detection of SHERP precedes that of HASPB. In contrast to earlier *in vitro* observations, however, loss of both proteins in the null parasites results in failure to produce metacyclics, decreased production of short promastigotes and lower colonization of the stomodeal valve (SV) region in late-stage infections in the sand fly. Conversely, complementation of the whole locus restores metacyclic production and SV colonization, while complementation with either HASPB alone or SHERP alone partially restores the wild-type phenotype. These data suggest that the HASP/SHERP proteins are critical for development of wild-type parasites in the sand fly and may therefore be essential in vector transmission.

## Results

### Expression of HASPB and SHERP during differentiation in culture

**Fig. 2:**
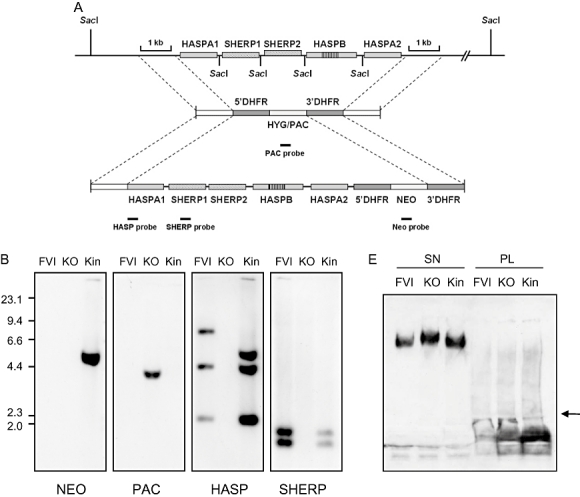

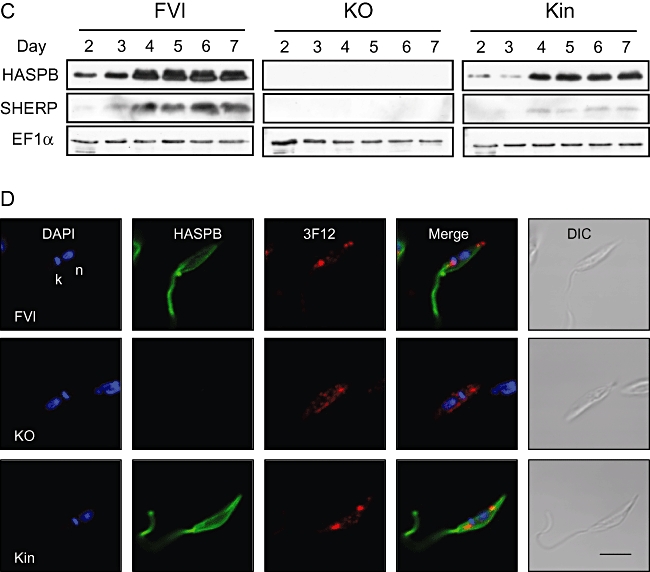
Targeted deletion and complementation of the LmcDNA16 locus.A. Schematic diagram of the LmcDNA16 locus and the plasmid constructs used for deletion and complementation. Flanking sequences used to generate the targeting vectors are shown. The top panel shows the LmcDNA16 locus, which contains two tandem identical copies of the *SHERP* gene, two non-tandem identical copies of the *HASPA* gene and a single copy of the *HASPB* gene. The *HASPB* open reading frame is highly similar to that of *HASPA* except for an additional repeat region (McKean *et al*., 1997a), indicated here by vertical line shading. The second panel shows the plasmid constructs used for targeted deletion of the locus as described previously (McKean *et al*., 2001) in which the locus was replaced by hygromycin/puromycin resistance genes (*HYG*/*PAC*). The third panel shows the plasmid construct used to produce a complemented parasite line in this study. This sequence contains flanking regions sharing identity with two sections of the deletion construct: the 5′ flanking region and the *DHFR* 3′UTR which is found downstream of the *HYG*/*PAC* genes. Correct integration of the complementation construct will replace either *HYG* or *PAC* with a single copy of the LmcDNA locus plus a copy of the neomycin resistance gene (*NEO*). Solid black bars represent fragments used as hybridization probes.B. Southern blot analysis of wild-type and mutant parasite lines. The following lines were analysed: wild type (FVI), *HYG*/*PAC* double replacement clone (ΔcDNA16::HYG/ΔcDNA16::PAC, KO) and a complemented double replacement clone in which the *PAC* gene had been replaced with a single copy of the cDNA16 locus (ΔcDNA16::HYG/ΔcDNA16::PAC/ΔPAC::cDNA16, Kin). Five micrograms of genomic DNA from each parasite line was digested with SacI, size separated through 0.8% agarose, blotted and hybridized with DIG-labelled DNA probes (∼200 bp) as indicated.C. Immunoblotting of wild-type and mutant parasite lines as in (B). Whole-cell lysates taken from parasites grown in culture for 2–7 days were separated by 10% (HASPB, EF1α) or 15% (SHERP) SDS-PAGE and immunoblots probed with polyclonal antisera raised against *L. major* HASPB and SHERP. A monoclonal antibody recognizing the constitutively expressed protein EF1α (clone CBP-KK1, Millipore) was used to confirm equivalent protein loading. Approximately 1 × 10^6^ parasites were loaded per lane.D. Expression of HASPB and LPG in metacyclic promastigotes of wild-type and mutant parasite lines (described in B and C). Parasites grown in culture for 7 days were agglutinated with peanut lectin and non-agglutinated cells fixed and permeabilized prior to indirect immunofluorescence analysis. DAPI (blue), nuclear and kinetoplast DNA; HASPB (green), 3F12 (red), antibodies specific for metacyclic LPG and HASPB respectively; DIC, differential interference contrast image. Size bar = 5 µm.E. Wild-type and mutant parasite lines (described in B and C) secrete proteophosphoglycans. Pellets prepared by ultracentrifugation of parasite culture supernatants (SN) or whole parasite lysates (PL) were separated by SDS-PAGE and blotted (arrow indicates boundary between 4% stacking gel and 12% resolving gel). The immunoblot was probed with monoclonal antibody LT6 (polyclonal rabbit antiserum against PPG; Ilg *et al*., 1993).

**Fig. 1:**
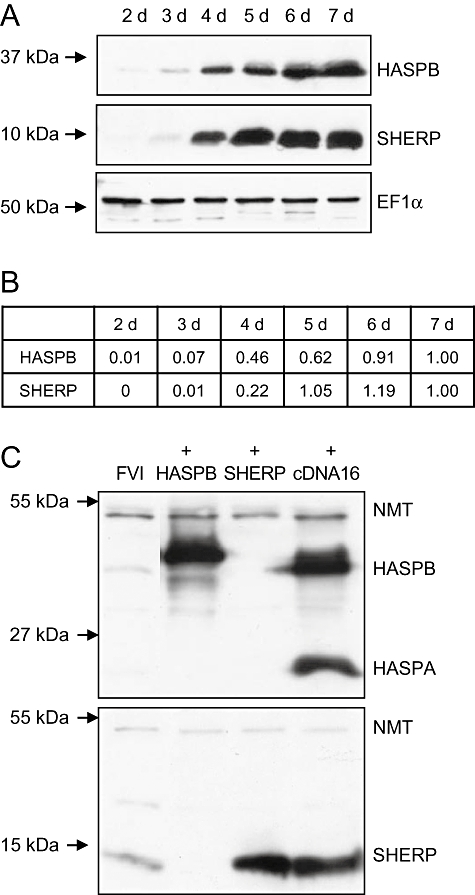
A. Expression of HASPB and SHERP during growth of *L. major* in culture. Immunoblot analysis of early passage wild-type parasites sampled over 7 days in culture. Whole-cell lysates (1 × 10^6^ parasites per track) were separated by SDS-PAGE and the gels immunoblotted with polyclonal antisera against HASPB (10% gel) or SHERP (15% gel). Probing with anti-EF1α (15% gel shown here) was used as an additional loading control.B. Densitometric analysis of HASPB/SHERP bands in (A) normalized to parasite numbers; values shown for each protein are relative to day 7.C. Expression of HASPB and SHERP complemented *L. major* after 3 days in culture. Samples were prepared as in (A) and the immunoblots (taken from 10% gels) probed with anti-HASPB (upper blot), anti-SHERP (lower blot) and anti-NMT (as a loading control for both blots). FVI, wild-type parasites; +HASPB, LmcDNA16 null complemented with pTEX HASPB; +SHERP, LmcDNA16 null complemented with pTEX SHERP; +cDNA16, LmcDNA16 null complemented with pTEX LmcDNA16 (all mutants are described in McKean *et al*., 2001). Molecular masses are indicated on the left of the blots.

HASPB and SHERP expression have been shown previously to correlate with parasite differentiation in culture, using mixed populations of promastigotes grown from log to stationary phase and sampled at fixed time points (Flinn *et al*., 1994; Knuepfer *et al*., 2001). To confirm the relative temporal expression of these two stage-regulated proteins, freshly differentiated *L. major* Friedlin promastigotes, derived from amastigotes isolated from the lymph nodes of susceptible mouse strains (as described in Depledge *et al*., 2009), were subject to minimum passage in culture prior to analysis over a time-course. Growth is not synchronized under these conditions, as shown by microscopic analysis of the relative numbers of procyclic, pre-metacyclic and metacyclic parasites over time (see *Experimental procedures*). Parasites were sampled at 24 h intervals from day 2 post-inoculation (when 99% of cells are procyclic) and total cell lysates (containing equivalent parasite numbers) analysed by immunoblotting with antibodies specific for HASPB and SHERP. As shown in Fig. 1A, both proteins show increased expression from day 4 (when 70% or cells are pre-metacyclic and 25% metacyclic) to day 7, when > 50% of viable parasites are of high motility, resistant to peanut lectin agglutination and can be classified as metacyclic promastigotes (see Fig. 2D). Comparison of the relative signals for the two proteins by densitometry, however, shows that SHERP expression is stabilized by 6 days while HASPB expression continues increasing to day 7 (Fig. 1B), suggesting that there are temporal differences in the expression of each molecule in these mixed parasite populations.

### Generation of new complemented lines for vector transmission studies

Previous analysis of genetically manipulated clones of *L. major* Friedlin either null for or complemented with the complete LmcDNA16 locus encoding HASP and SHERP genes failed to show a phenotype distinct to wild-type parasites in culture or after macrophage infection *in vitro* and *in vivo* (McKean *et al*., 2001). In these experiments, the ‘add-back’ parasites were avirulent, an observation interpreted as an overexpression phenotype due to the excessive amounts, and loss of regulated expression, of the HASP and SHERP proteins from the complementing plasmid. In Fig. 1C, comparing lysates of log-phase promastigotes of wild-type *L. major* Friedlin (FVI) with the three complemented lines (+HASPB, +SHERP and +cDNA16) described in McKean *et al*. (2001) after 3 days in culture, it is evident that both HASPB and SHERP are overexpressed in the single add-back lines. Similarly, the open reading frames of the LmcDNA16 locus (HASPB, HASPA and SHERP) are all overexpressed following complementation with the complete locus as compared with the wild-type parasites, in which only low levels of SHERP and the HASPs are detectable at this stage of the parasite life cycle (Fig. 1C). Before proceeding to sand fly transmission experiments therefore we generated a new complemented line expressing a single copy of the LmcDNA16 locus, coupled with a constitutively expressed *NEO* gene reintroduced into one allele of the original locus, thereby generating a heterozygous add-back parasite line. Correct genomic integration of the LmcDNA16 complementation cassette (Fig. 2A) was confirmed by Southern blot analysis (Fig. 2B). In the clones analysed here, the *NEO* probe hybridizes to a single fragment of 4.8 kb in the complemented double replacement clone (Kin) and this is absent from wild-type (FVI) and null (KO) parasites. The *PAC* gene is found on a single fragment of 3.8 kb in the null clone but is absent as expected in wild-type DNA. The *PAC* gene is also absent from the Kin clone, demonstrating that the *PAC* cassette has been replaced with *NEO* in these cells. As expected, neither *HASPB* nor *SHERP* is detected in the null clone. The *HASP* probe hybridizes in wild-type cells to fragments of 7.6 kb, 4.3 kb and 2.2 kb, corresponding to the expected sizes of *HASPA2*, *HASPA1* and *HASPB* respectively. The 4.3 kb and 2.2 kb bands (*HASPA1* and *HASPB*) are also seen in the Kin clone, together with a band of 4.8 kb representing *HASPA2* plus the inserted NEO gene. The *SHERP* probe is detected as two bands of 1.8 kb and 1.6 kb (corresponding to *SHERP1* and *SHERP2* respectively*)* in both the wild-type and Kin clone. These data verify the genetic structure of the inserted add back (which has subsequently been confirmed by DNA sequencing and comparison to GenBank entry AJ237587.1).

Immunoblotting of lysates taken from the Kin line and wild-type parasites collected over a 7-day time-course was used to verify regulated expression of the HASPB and SHERP proteins in the complemented Kin parasites. As shown in the clones analysed in Fig. 2C, a similar temporal pattern of expression of HASPB and SHERP is detected in both wild-type and add-back cells, although the relative levels of expression are lower in the Kin parasites, as would be expected in a heterozygous add-back line.

To correlate these observations with expression specifically in metacyclic stages, parasites of the Kin and null lines, together with wild-type cells, were grown to 7 days in culture and then agglutinated with peanut lectin. Non-agglutinated parasites (defined as metacyclics in culture and in infected sandflies using the metacyclic LPG-specific antibody, 3F12; Sacks and da Silva, 1987; Saraiva *et al*., 1995) were then analysed by indirect immunofluorescence using confocal microscopy. As shown in Fig. 2D, non-agglutinated parasites from all three lines have the typical metacyclic morphology of short body and relatively long flagellum. As a consequence, the nucleus and kinetoplast are located close together, as detected by DAPI staining of their DNA. All three lines are also cross-reactive with 3F12, which produces a punctuate staining pattern under the fixation conditions used with confocal microscopy. Only the wild-type and Kin parasites express HASPB, as expected. These observations using a defined metacyclic LPG marker confirm that the null parasites can undergo metacyclogenesis as reported previously (McKean *et al*., 2001).

To further characterize and compare the phenotype of the new Kin line with the null and wild-type parasites (independently from detection of either HASPB or metacyclic LPG), total glycoconjugate production was monitored in parasite lysates and secretory fractions of late-log-phase parasites (day 5 post-inoculation). The antibody used for detection, LT6 (Ilg *et al*., 1993), recognizes the galactose-mannose-phosphate disaccharide repeat units of LPG and secreted proteophosphoglycan (PPG; Rogers *et al*., 2004). As shown in Fig. 2E, LPG is detected in total lysates from wild-type, KO and Kin parasites while secreted PPG is also abundant in the culture supernatant from all three lines. While this analysis would not detect structural differences between the different glycoconjugate fractions, these data demonstrate that PPG secretion is not compromised following deletion of the LmcDNA16 locus, at least *in vitro*. *In vivo*, PPG is secreted by short (leptomonad) promastigotes in *Leishmania mexicana* (Rogers *et al*., 2002), some of which differentiate into metacyclic parasites primed for transmission (Bates, 2007). Our observation of PPG secretion by the KO *L. major* parasites described above suggests that *in vitro* differentiation can generate elongated nectomonads and short promastigotes in this line.

### Development of LmcDNA16 mutant parasites in *P. papatasi*

To investigate a potential role for the HASP and SHERP genes in vector transmission, we used wild-type promastigotes of *L. major* Friedlin and the three mutant lines described in McKean *et al*. (2001): *Lmc*DNA16 double-knockout or null (KO; *ΔcDNA16::HYG/ΔcDNA16::PAC*), *Lmc*DNA16 double-knockout complemented with episomal HASPB (+HASPB; *ΔcDNA16::HYG/ΔcDNA16::PAC [pTEX NEO HASPB]*, as used in Fig. 1C) and *Lmc*DNA16 double-knockout complemented with episomal SHERP (+SHERP; *ΔcDNA16::HYG/ΔcDNA16::PAC [pTEX NEO SHERP]*, as used in Fig. 1C). In addition, the new add-back line described above, *ΔcDNA16::HYG/ΔcDNA16::PAC/ΔPAC::cDNA16* (Kin), was used instead of the original episomally complemented parasites analysed in Fig. 1C (+cDNA16; McKean *et al*., 2001). Female sand flies were infected by feeding through a chick-skin membrane on heat-inactivated rabbit blood containing promastigotes of each parasite line. Engorged sand flies were dissected over a time-course post-blood meal (PBM) and the location and rates of infection in the digestive tract determined by dissection and light microscopy. Parasite loads were also confirmed by quantitative PCR (qPCR) analysis of dissected guts.

**Fig. 4:**
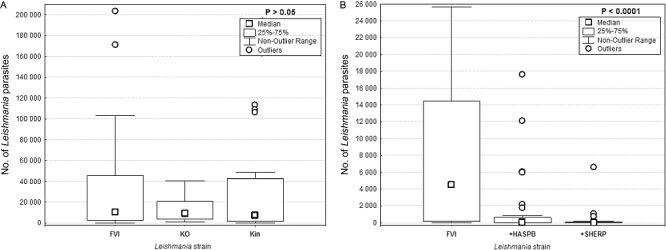
Analysis of parasite loads in sand flies infected with the lines described in Fig. 3. qPCR was used to determine parasite DNA content in midguts from females with late-stage infections (10–12 days PBM). Thirty to 40 midguts were analysed for each line.A. Non-significant differences among FVI, KO and Kin parasites in *P. papatasi*, *P* = 0.8796, anova, Kruskal–Wallis test.B. Significant differences among FVI, +SHERP and +HASPB parasites in *P. papatasi*, *P* < 0.0001, anova, Kruskal–Wallis test.

**Fig. 3:**
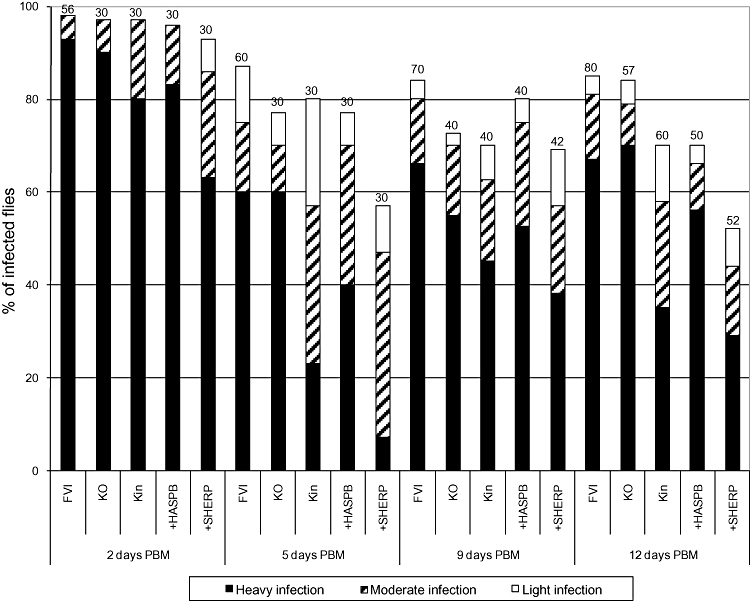
Rates and intensities of infections with five *L. major* lines in *P. papatasi*. The parasite lines analysed were as follows: FVI, *L. major* Friedlin wild type; KO, ΔcDNA16::HYG/ΔcDNA16::PAC; Kin, ΔcDNA16::HYG/ΔcDNA16::PAC/ΔPAC::cDNA16; +HASPB, as in Fig. 1C; +SHERP, as in Fig. 1C. Sand fly females were infected by feeding through a chick-skin membrane on a suspension of 10^6^ parasites ml^−1^ of rabbit blood and dissected 2, 5, 9 and 12 days PBM. Gut infections were graded as light (< 100 parasites per gut), moderate (100–1000 parasites per gut) or heavy (> 1000 parasites per gut) as described in *Experimental procedures*. Infection experiments were repeated four times for combinations of wild-type (FVI), KO and Kin lines and twice for combinations of FVI, +HASPB and +SHERP lines. Differences between lines were evaluated using the chi-square test: day 2 PBM, *P* = 0.068, χ^2^ = 19.953, d.f. = 12; day 5 PBM, *P* < 0.0001, χ^2^ = 40.657, d.f. = 12; day 9 PBM, *P* = 0.344, χ^2^ = 13.357, d.f. = 12; day 12 PBM, *P* < 0.0001, χ^2^ = 41.544, d.f. = 12. Numbers above each bar, number of flies analysed.

Dissections of these sand fly females showed that all *L. major* lines were able to develop heavy infections in *P. papatasi*. However, quantitative differences in parasite load between the lines occurred after defecation on day 5 PBM and on day 12 (Fig. 3). Results of qPCR analysis revealed that differences between the wild-type strain (FVI), double-knockout (KO) and complemented (Kin) lines in parasite loads were not significant in late-stage infections (Fig. 4A). However, infections with +HASPB were significantly less abundant than wild-type infections (*P* = 0.0005, *U* = 197.0, *Z* = 3.470) while +SHERP infections were even less abundant than +HASPB infections (*P* = 0.0339, *U* = 306.5, *Z* = 2.122; Fig. 4B).

After escape from the peritrophic matrix, *L. major* development within the midgut of *P. papatasi* involves transformation of elongated nectomonads to short promastigotes (leptomonads in the terminology of Rogers *et al*., 2002) and highly motile metacyclic forms. Parasites migrate to the thoracic part of the midgut and colonize the SV; forms that attach to the SV are called haptomonads. In the experiments described in Table 1, significant differences were observed in the location of infections within the infected sand flies. Wild-type (FVI), complemented (Kin) and +HASPB lines showed heavy colonization of the SV with many haptomonads firmly adhering to its chitinous lining. Conversely, null parasites (KO) did not colonize the SV (only one female from 97 dissected by days 9 and 12 PBM had weak colonization of the SV), although the anterior part of the thoracic midgut near the SV, named the cardia, was reached by elongated nectomonads quite early after infective feeding (57% of infected flies by day 5 PBM). The +SHERP parasites colonized the SV in a small percentage of infected sand flies; only nine females from 82 dissected by 9 and 12 days PBM had short promastigotes weakly attached to the SV. Similarly to the null line, the parasite population of the +SHERP line consisted mostly of elongated nectomonads.

**Table 1:** Location of parasites in *P. papatasi* infected with the lines described in Fig. 3.

Days PBM	*Leishmania* line	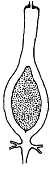 Endoperitrophic space	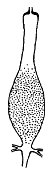 AMG only	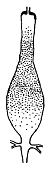 AMG and TMG	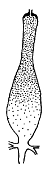 AMG, TMG and cardia	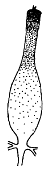 Colonized SV
2	FVI	100	0	0	0	0
	KO	100	0	0	0	0
	Kin	100	0	0	0	0
	+HASPB	100	0	0	0	0
	+SHERP	100	0	0	0	0
5	FVI	0	11.5	42.3	44.2	1.9
	KO	0	8.7	34.8	56.5	0.0
	Kin	0	20.8	45.8	33.3	0.0
	+HASPB	0	26.1	30.4	43.5	0.0
	+SHERP	0	17.6	47.1	35.3	0.0
9	FVI	0	5.1	13.6	39.0	42.4
	KO	0	3.4	6.9	89.7	0.0
	Kin	0	17.9	10.7	50.0	21.4
	+HASPB	0	3.1	15.6	46.9	34.4
	+SHERP	0	3.4	37.9	48.3	10.3
12	FVI	0	4.4	4.4	30.9	60.3
	KO	0	6.3	18.8	72.9	2.1
	Kin	0	14.3	9.5	38.1	38.1
	+HASPB	0	2.9	5.7	34.3	57.1
	+SHERP	0	0.0	18.5	59.3	22.2

*Leishmania* infections in the sand fly digestive tract at 2, 5, 9 and 12 days PBM were analysed by dissection and examination by light microscopy. The % of infected flies found in each location is shown. AMG, abdominal midgut; TMG, thoracic midgut; SV, stomodeal valve.

**Fig. 5:**
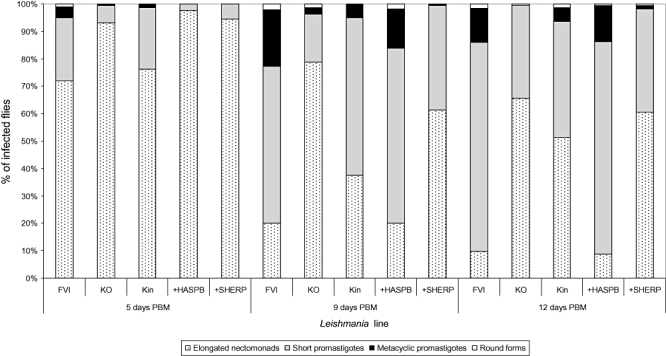
Morphological forms of the lines described in Fig. 3 during development in *P. papatasi*. The guts of *L. major* infected females were sampled at 5, 9 and 12 days PBM and parasite morphometry determined as described (*Experimental procedures*). The % of each form found in infected flies at each time point is shown. Differences among lines were significant and increased from day 5 PBM (*P* < 0.0001, χ^2^ = 93.266, d.f. = 12) to day 9 PBM (*P* < 0.0001, χ^2^ = 246.947, d.f. = 12) and day 12 PBM (*P* < 0.0001, χ^2^ = 283.947, d.f. = 12).

Differences between the five *L. major* lines in the presence and size of the four morphological categories (elongated nectomonads, short promastigotes, metacyclic promastigotes and round forms) were significant at all time intervals tested (Fig. 5, Table 2). However, the following similarities between lines were observed: (i) the FVI and +HASPB lines had shorter bodies and higher proportions of short and metacyclic promastigotes then the other three lines at 9 and 12 days PBM, (ii) the FVI and Kin lines had shorter bodies and lower numbers of elongated nectomonads than the other three lines at 5 days PBM, and (iii) the KO and +SHERP lines had longer bodies and higher proportions of elongated nectomonads than the other lines at 9 and 12 days PBM.

**Table 2:** Dimensions of the morphological forms of the lines described in Fig. 3 during development in *P. papatasi* at 2, 5, 9 and 12 days PBM.

				Body length		Body width		Flagellar length	
Days PBM	*Leishmania* strain	Morphological form	*n*	Mean (SD) (µm)	Range (µm)	Mean (SD) (µm)	Range (µm)	Mean (SD) (µm)	Range (µm)
5	FVI	EN	230	19.0(3.3)	14.9–34.5	1.5(0.5)	0.7–3.3	18.3(2.4)	13.3–25.5
		SP	74	11.5(1.9)	5.0–13.5	1.9(0.6)	0.7–4.5	14.4(2.9)	5.0–19.9
		MP	13	8.1(1.6)	5.0–9.9	1.6(0.1)	1.5–1.7	17.8(2.4)	14.9–21.6
		RF	3	4.4(0.9)	3.3–5.0	2.2(0.9)	1.6–3.3	13.8(3.5)	10.0–16.6
		**Total**	**320**	**16.7(4.8)**	**3.3–34.5**	**1.6(0.6)**	**0.7–4.5**	**17.3(3.0)**	**5.0–25.5**
	KO	EN	149	19.7(3.4)	15.0–30.0	1.4(0.3)	0.7–1.5	17.2(2.4)	12.0–25.5
		SP	10	12.1(1.1)	10.5–13.5	1.5(0.0)	1.5–1.5	13.5(2.5)	9.0–18.0
		MP	1	4.5		1.5		15.0	
		**Total**	**160**	**19.1(3.9)**	**4.5–30.0**	**1.4(0.3)**	**0.7–1.5**	**17.0(2.6)**	**9.0–25.5**
	Kin	EN	122	18.3(2.4)	15.0–25.5	1.4(0.5)	0.7–3.0	17.1(2.2)	9.0–24.0
		SP	35	12.3(1.3)	9.0–13.5	1.7(0.6)	0.7–3.0	14.1(2.6)	9.0–21.0
		MP	2	8.25(1.0)	7.5–9.0	1.1(0.5)	0.7–1.5	18.7(1.1)	18.0–19.5
		**Total**	**160**	**16.8(3.6)**	**7.5–25.5**	**1.4(0.5)**	**0.7–3.0**	**16.4(2.6)**	**9.0–24.0**
	+HASPB	EN	156	20.6(2.9)	14.9–28.2	1.7(0.3)	0.8–3.3	20.2(2.3)	14.9–26.6
		SP	4	11.2(1.6)	9.9–13.3	1.7(0.0)	1.7–1.7	16.2(5.5)	10.0–21.6
		**Total**	**160**	**20.4(3.2)**	**9.9–28.2**	**1.7(0.3)**	**0.8–3.3**	**20.1(2.5)**	**10.0–26.6**
	+SHERP	EN	151	19.0(2.4)	14.9–24.9	1.7(0.4)	0.8–3.3	18.3(2.0)	13.3–24.9
		SP	9	11.8(2.2)	8.3–13.3	1.7(0.0)	1.7–1.7	15.5(2.9)	13.3–19.9
		**Total**	**160**	**18.6(2.9)**	**8.3–24.9**	**1.7(0.4)**	**0.8–3.3**	**18.1(2.2)**	**13.3–24.9**
9	FVI	EN	64	16.7(2.6)	15.0–25.5	1.6(0.4)	0.7–3.0	17.0(2.5)	13.5–24.0
		SP	183	10.6(2.2)	4.5–13.5	1.6(0.5)	0.7–3.3	13.3(2.9)	4.5–21.6
		MP	66	7.3(1.7)	4.5–12.0	1.5(0.4)	0.7–3.0	16.3(3.3)	10.0–24.0
		RF	7	4.0(0.7)	3.3–5.0	4.1(0.7)	3.3–5.0	14.1(5.3)	8.3–24.9
		**Total**	**320**	**11.0(3.9)**	**3.3–25.5**	**1.7(0.6)**	**0.7–5.0**	**14.7(3.4)**	**4.5–24.9**
	KO	EN	126	18.6(3.6)	15.0–30.0	1.5(0.3)	0.7–3.0	18.5(3.3)	12.0–27.0
		SP	28	10.9(1.8)	6.0–13.5	2.2(1.0)	0.7–4.5	14.1(4.3)	4.5–22.5
		MP	4	9.7(1.5)	7.5–10.5	2.2(0.9)	1.5–3.0	19.9(3.3)	15.0–22.5
		RF	2	4.5(0.0)	4.5–4.5	4.5(0.0)	4.5–4.5	12.7(7.4)	7.5–18.0
		**Total**	**160**	**16.9(4.8)**	**4.5–30.0**	**1.7(0.7)**	**0.7–4.5**	**17.7(3.9)**	**4.5–27.0**
	Kin	EN	60	17.1(2.7)	15.0–24.0	1.6(0.5)	0.7–3.0	15.8(2.4)	10.5–22.5
		SP	92	10.8(2.0)	4.5–13.5	1.6(0.7)	0.7–4.5	12.6(2.1)	7.5–19.5
		MP	8	7.9(1.6)	6.0–10.5	1.5(0.0)	1.5–1.5	17.2(3.0)	12.0–22.5
		**Total**	**160**	**13.0(3.9)**	**4.5–24.0**	**1.6(0.6)**	**0.7–4.5**	**14.0(2.8)**	**7.5–22.5**
	+HASPB	EN	32	16.0(0.8)	14.9–16.6	1.6(0.4)	0.8–2.5	16.8(2.0)	11.6–19.9
		SP	102	11.0(1.8)	6.6–13.3	1.6(0.3)	0.8–3.3	13.4(2.2)	6.6–19.9
		MP	23	7.4(1.4)	5.0–10.0	1.7(0.0)	1.7–1.7	15.8(2.2)	10.0–19.9
		RF	3	5.0(2.9)	3.3–8.3	3.9(0.9)	3.3–5.0	7.2(2.5)	5.0–10.0
		**Total**	**160**	**11.4(3.1)**	**3.3–16.6**	**1.6(0.5)**	**0.8–5.0**	**14.3(2.8)**	**5.0–19.9**
	+SHERP	EN	98	19.4(2.9)	14.9–29.9	1.6(0.4)	0.8–3.3	19.1(2.8)	13.3–30.0
		SP	61	11.1(1.8)	8.3–13.3	1.5(0.4)	0.8–2.5	13.9(2.5)	6.6–19.9
		MP	1	8.3		0.8		16.6	
		**Total**	**160**	**16.2(4.8)**	**8.3–29.9**	**1.6(0.4)**	**0.8–3.3**	**17.1(3.7)**	**6.6–30.0**
12	FVI	EN	31	15.6(1.0)	15.0–18.3	1.9(0.7)	0.7–4.5	14.9(2.2)	10.5–19.5
		SP	244	9.9(2.2)	4.5–13.5	1.8(0.8)	0.7–6.0	11.3(2.9)	0.0–18.3
		MP	40	6.6(1.4)	4.5–9.0	1.6(0.5)	0.8–3.3	15.1(3.3)	9.0–22.5
		RF	5	6.5(1.4)	5.0–7.5	5.0(0.6)	4.5–6.0	5.8(5.5)	0.0–11.6
		**Total**	**320**	**10.0(2.9)**	**4.5–18.3**	**1.8(0.9)**	**0.7–6.0**	**12.0(3.4)**	**0.0–22.5**
	KO	EN	105	17.2(2.3)	15.0–22.5	1.6(0.6)	0.7–3.0	15.1(2.3)	9.0–22.5
		SP	54	11.7(1.7)	7.5–13.5	1.9(0.8)	0.7–3.0	11.9(3.1)	6.0–21.0
		RF	1	7.5		4.5		9.0	
		**Total**	**160**	**15.3(3.4)**	**7.5–22.5**	**1.7(0.7)**	**0.7–4.5**	**14.0(3.0)**	**6.0–22.5**
	Kin	EN	82	19.2(4.1)	14.9–28.2	1.7(0.4)	0.8–3.3	18.7(4.5)	11.6–28.2
		SP	68	11.9(1.7)	6.6–13.3	1.9(0.6)	0.8–3.3	13.4(2.4)	8.3–19.9
		MP	8	8.3(1.5)	5.0–10.0	2.2(0.8)	1.7–3.3	17.6(4.2)	10.0–24.9
		RF	2	8.3(4.7)	5.0–11.6	5.0(0.0)	5.0–5.0	16.6(2.3)	14.9–18.3
		**Total**	**160**	**15.4(5.0)**	**5.0–28.3**	**1.8(0.6)**	**0.8–5.0**	**16.4(4.5)**	**8.3–28.2**
	+HASPB	EN	14	15.9(1.1)	15.0–18.3	1.9(0.6)	0.8–3.3	15.8(1.1)	14.9–18.3
		SP	124	10.6(2.0)	6.6–13.3	1.7(0.4)	0.8–3.3	13.0(2.4)	6.6–19.9
		MP	21	7.1(1.5)	5.0–10.0	1.8(0.5)	1.7–3.3	14.9(2.5)	10.0–19.9
		RF	1	5.0		3.3		8.3	
		**Total**	**160**	**10.6(2.8)**	**5.0–18.3**	**1.7(0.5)**	**0.8–3.3**	**13.4(2.6)**	**6.6–19.9**
	+SHERP	EN	97	18.2(2.1)	14.9–23.2	1.8(0.4)	0.8–3.3	17.8(2.1)	11.6–23.2
		SP	60	11.3(1.9)	8.3–13.3	1.7(0.6)	0.8–3.3	13.0(2.4)	8.3–19.9
		MP	2	5.0(0.0)	5.0–5.0	2.5(1.2)	1.7–3.3	11.6(2.3)	10.0–13.3
		RF	1	6.6		6.6		16.6	
		**Total**	**160**	**15.4(4.2)**	**5.0–23.2**	**1.8(0.6)**	**0.8–6.6**	**15.9(3.2)**	**8.3–23.2**

Promastigotes from gut smears were measured by light microscopy with an oil-immersion objective.

EN, elongated nectomonads; SP, short promastigotes; MP, metacyclic promastigotes; RF, round forms.

Although all five *L. major* lines were able to develop heavy late-stage infections in *P. papatasi*, the observed differences in both the locations of the infections in the fly and the parasite morphology allows the division of the tested lines into two groups. The first of these is composed of the wild-type FVI strain and the Kin and +HASPB lines, which all showed classical *Leishmania* development with transformation of elongated nectomonads to short promastigotes and metacyclic promastigotes and colonization of the SV in late-stage infections. Parasites of the second group, consisting of null and +SHERP mutants, remained mostly in the stage of elongated nectomonads and colonized the abdominal and thoracic midgut but not the SV.

**Fig. 7:**
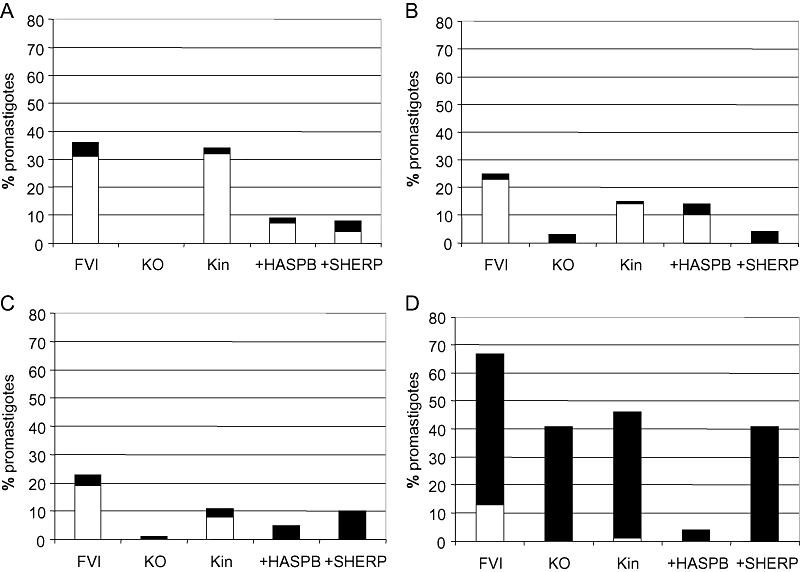
Expression of HASPB, SHERP and metacyclic LPG in promastigote stages of the parasite lines described in Fig. 3. Gut smears obtained by dissection of infected sandflies at day 9 and 12 PBM were analysed by indirect immunofluorescence as described in *Experimental procedures*. Promastigotes were measured and their reaction with antibodies was evaluated using Image J software. The % of metacyclic promastigotes of each line is shown, staining either positive (open box) or negative (closed box) with (A) 3F12 antibody for LPG; (B) anti-HASPB; (C) anti-SHERP. Recognition of short promastigotes with anti-SHERP is shown in (D). In all cases (A–D), the differences between strains were significant, as measured by chi-square test. [(A) χ^2^ = 10.339, d.f. = 3, *P* = 0.016, (B) χ^2^ = 29.160, d.f. = 4, *P* < 0.0001, (C) χ^2^ = 27.914, d.f. = 4, *P* < 0.0001, (D) χ^2^ = 23.840, d.f. = 4, *P* < 0.0001.]

**Fig. 6:**
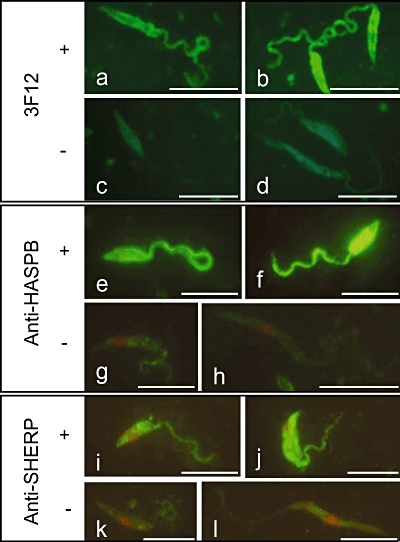
Expression of HASPB, SHERP and metacyclic LPG in promastigote stages of *L. major* FVI dissected from *P. papatasi* at 12 days PBM. Indirect immunofluorescence analysis was carried out with antibodies specific for each marker: a–d, 3F12 antibody for metacyclic LPG; e–h, anti-HASPB; i–l, anti-SHERP. Images a, b: 3F12-positive metacyclic promastigotes (MP); image c: 3F12-negative short promastigote (SP); image d: 3F12-negative SP and elongated nectomonad (EN); images e, f: HASPB-positive MPs; image g: HASPB-negative SP; image h: HASPB-negative EN; image i: SHERP-positive MP; image j, SHERP-positive SP; image k, SHERP-negative SP; image l, SHERP-negative EN. Size bars = 10 µm.

To correlate the identity of parasite stages defined by morphology in the sand fly with expression of stage-specific markers, antibodies specific for HASPB, SHERP and metacyclic LPG were used for indirect immunofluorescence microscopy. The stage specificity of these reagents in the sand fly is illustrated in Fig. 6 which shows their reactions with wild-type *L. major* dissected from infected flies in late-stage infections. Staining with the 3F12 antibody specific for metacyclic LPG (Sacks and da Silva, 1987) identified metacyclic promastigotes only, with no recognition of short promastigotes or elongated nectomonads (Fig. 6, images a–d). Similarly, anti-HASPB also showed specificity for metacyclic parasites and no cross-reactivity with the previous developmental stages (Fig. 6, images e–h). Conversely, while anti-SHERP also identified metacyclics, it also cross-reacted with short promastigotes but not with elongated nectomonads (Fig. 6, images i–l). With this knowledge, the same antibodies were used to monitor how the frequency of the various morphological forms differed between smears taken from individual flies (Fig. 7).

At 5 days PBM, no positive reaction was detected with either the HASPB or SHERP antibodies (results not shown). In all *Leishmania* lines at later stages of infection (9 or 12 days PBM), 3F12 and anti-HASPB reacted only with parasite stages identified morphologically as metacyclics (Fig. 7A and B), as described above for the wild-type strain. Conversely, SHERP antibodies reacted not only with metacyclics but also with some short promastigotes of the wild-type strain and Kin line (Fig. 7C and D). These positively staining parasites may represent some intermediate forms with flagella not long enough to allow identification as metacyclic cells. Generally, the highest percentage of promastigotes morphologically identified as metacyclics was found in the wild-type FVI strain (23–36%) and the lowest in the null line (0–3%). The metacyclic LPG antibody, 3F12, positively identified metacyclic parasites in four of the five lines tested at 9 or 12 days PBM but no metacyclics were found in the null parasite infections. Anti-HASPB reacted positively with metacyclic parasites of three lines (FVI, +HASPB and Kin) but not with the null and +SHERP lines, as expected. Negative reaction of anti-SHERP antibodies was observed with KO and +HASPB lines as expected but, surprisingly, also with parasites of the +SHERP line, both as metacyclics and as short promastigotes. These results suggest not only that SHERP function may be required at an earlier stage than HASPB function but also that HASPB expression may be required for SHERP activity *in vivo*.

## Discussion

In this article, we reveal a novel role for two previously described *Leishmania* proteins that show stage-specific expression, at both the RNA and protein level, during the parasite life cycle (Flinn and Smith, 1992; Flinn *et al*., 1994; Knuepfer *et al*., 2001). As originally demonstrated under *in vitro* culture conditions, and here confirmed during development in *P. papatasi*, the HASPB and SHERP proteins are highly upregulated in infective metacyclic stages, with SHERP also showing low-level expression in the preceding short promastigote stage in the vector. Neither protein is detectable at earlier developmental stages in the sand fly, definitively confirming the stage-specific expression of these molecules. Low-level HASPB and SHERP signal can be observed in logarithmic phase culture due to a lack of synchrony in parasite growth and differentiation and the presence of a mixture of parasite stages in the starting inoculum. These practical issues are partially resolved by using low-passage parasites that exhibit higher expression levels of both proteins (see Fig. 1A). Under these conditions, SHERP expression peaks before maximum HASPB expression, likely correlating with its detection in short promastigotes although these cannot be definitively identified in culture.

The observations above confirm the utility of HASPB as a marker for metacyclogenesis in *L. major* and suggest a vital role for both HASPB and SHERP either in this differentiation process and/or in the metacyclic parasite. HASPB in *L. major* is transported to the plasma membrane, a process requiring N-terminal modification byacylation (Denny *et al*., 2000). At this location, it can be detected externally on both the cell body and flagellum, most recently by imaging in live parasites (L. Maclean, unpublished). Our current model proposes that HASPB can be shed on macrophage entry, a process that could optimize presentation to the host immune system. Recombinant *Leishmania donovani* HASPB is a target for recognition by B-1 B cell-derived natural antibodies, with the resulting immune complexes triggering classical complement pathway activation, leading to IL-4 secretion, CD8+ T cell priming and vaccination against parasite infection in immuno-compromised mice (Stager *et al*., 2003). Whether HASPB is shed from metacyclics in the vector has not yet been established.

SHERP, in contrast to HASPB, is a peripheral membrane protein that is not expressed by parasites in the host. In wild-type promastigotes, SHERP is localized to the cytosolic face of the ER and the outer mitochondrial membrane (Knuepfer *et al*., 2001), with recent biochemical and structural analysis suggesting that membrane lipid interactions may drive the function of this unusual small protein (B. Moore, unpublished). Another potential focus for SHERP interactions is with the vacuolar ATPase protein complex, a membrane-localized pump that drives proton transport across the eukaryotic plasma membrane but also functions in acidification of subcellular compartments, including those within the endosomal/lysosomal system (Sun-Wada *et al*., 2004). This may be of particular significance in metacyclic parasites given the recent identification of autophagy as a key process in parasite differentiation and virulence (Besteiro *et al*., 2006; 2007;). In cultured *L. major*, dividing promastigotes are characterized by a multivesicular body-like network that matures into a lysosomal-like structure of high lytic capacity and low pH in metacyclic parasites. Given SHERP's intracellular localization to ER and mitochondrial membranes in *L. major* (Knuepfer *et al*., 2001) and the potential for both membrane types to be processed by autophagic digestion, perhaps SHERP plays a regulatory role in vacuolar acidification during autophagy in the vector.

Genetic analysis of parasite mutants has been critical for the analysis and confirmation of gene function in *Leishmania* species (e.g. Spath *et al*., 2003; Ortiz *et al*., 2007). However, parasites deleted for the LmcDNA16 locus containing HASP and SHERP genes failed to present a phenotype distinct from wild-type parasites when used directly to infect either cultured macrophages or susceptible BALB/c mice *in vivo*, while ‘add-back’ clones yielded phenotypic features consistent with protein overexpression (McKean *et al*., 2001). Of particular note, the null parasites were able to undergo metacyclogenesis *in vitro*, as monitored by analysis of stage-specific transcript patterns and resistance to agglutination with peanut lectin. These observations suggested that the products of the HASP/SHERP locus did not play a role in parasite differentiation (McKean *et al*., 2001). Importantly, the mouse infection experiments were initiated by high-dose needle injection of metacyclic-rich parasite populations rather than by experimental inoculation by sand fly bite, an approach that mimics parasite transmission *in vivo*.

Results presented in this article confirm that the LmcDNA16 null parasites described above can differentiate into metacyclics *in vitro*, as defined by expression of the 3F12 epitope on metacyclic LPG. We have now developed a new complemented Kin line expressing HASPB and SHERP proteins at similar levels to wild-type parasites and, importantly, at the correct stage of development. These parasites, together with the null line, have been used to infect female *P. papatasi* and parasite growth and differentiation measured following blood meal digestion, using both morphological and biochemical markers. The results of these analyses demonstrate conclusively that the genetic locus encoding HASPB and SHERP is essential for metacyclogenesis in the sand fly: null parasites accumulate at the earlier elongated nectomonad stage of development and do not colonize the SV. As a result, we predict that the null mutants cannot be transmitted to the host by sand fly bite since destruction of the vector SV has been described to facilitate the transmission of *Leishmania* and *Trypanosoma* parasites (Volf *et al*., 2004).

These observations, demonstrating that the LmcDNA16 null parasites can undergo metacyclogenesis *in vitro* but not *in vivo*, suggest either that the metacyclic phenotype observed in the vector and required for parasite transmission *in vivo* is not fully replicated *in vitro* or that other factors are critical for differentiation in the sand fly. Perhaps the loss of proteins expressed from this locus impacts on parasite adhesion, migration or sensitivity to midgut hydrolases or, alternatively, plays a role in establishment at the SV. Is the null phenotype observed in sand flies caused by the lack of HASPB and/or SHERP function? And what is the role of the HASPA genes (McKean *et al*., 1997b) that are also present in the deleted LmcDNA16 locus and code for HASP proteins lacking the central repetitive domain of HASPB?

Using the single gene complemented lines (+HASPB and +SHERP), we have shown that HASPB alone can complement the null phenotype observed with the KO parasites, with these parasites able to complete classical development and colonize the SV in late-stage infections. However, it should be noted that +HASPB parasites overexpress the protein constitutively, a property shared by the +SHERP line with respect to SHERP expression. We therefore conclude that while HASPB is likely to be the dominant molecule in restoring the null phenotype, modulation of HASPB function by other factors in metacyclic parasites is not ruled out by these observations. Clearly, SHERP expression precedes HASPB expression and it is possible that the functions of these two proteins are linked during metacyclogenesis. An additional role for the HASPA proteins in this process cannot be discounted.

Delineating the function of all genes within the LmcDNA16 locus and their relative roles in parasite transmission will require an extended study utilizing a range of new transgenic lines expressing each gene product appropriately, both quantitatively and temporally. These approaches may reveal mechanistic details of metacyclogenesis within the sand fly and, as a consequence, underpin our understanding of transmission of the *Leishmania* parasite *in vivo*.

## Experimental procedures

### Parasites

*Leishmania major* Friedlin (MHOM/IL/81/Friedlin/VI; FVI) and mutant parasites targeted for gene disruption/overexpression at the LmcDNA16 locus were maintained on blood agar slopes or in culture as previously described (McKean *et al*., 2001). Low-passage promastigotes (2–3 cycles post transformation from mouse-derived amastigotes) were inoculated at 1 × 10^5^ ml^−1^ and the culture sampled daily. Parasite numbers, morphology and motility were monitored by light microscopy, counting > 100 parasites per line per time point and scoring for metacyclics (small body, flagellum ∼2× body length, high motility), pre-metacyclics (heterogeneous population of parasites showing shortening body and moderate motility) and procyclics (extended cell body ∼2× that of metacyclic parasites with relatively short flagellum).

The mutant lines used were the 4.8 *Lmc*DNA16 double-knockout (KO; *ΔcDNA16::HYG/ΔcDNA16::PAC*), the 4.8 *Lmc*DNA16 double-knockout complemented with episomal HASPB (+HASP; *ΔcDNA16::HYG/ΔcDNA16::PAC [pTEX NEO HASPB]*) and the 4.8 *Lmc*DNA16 double-knockout complemented with episomal SHERP (+SHERP; *ΔcDNA16::HYG/ΔcDNA16::PAC [pTEX NEO SHERP];*McKean *et al*., 2001).

A new complemented (knock-in, Kin) LmcDNA16 line was generated as described in Fig. 2, by introduction of a linear fragment containing the complete LmcDNA16 locus plus constitutively expressed *NEO* gene into its original location on chromosome 23 in a heterozygous (LmcDNA16 locus single deletion) background, using homologous recombination as described in McKean *et al*. (2001). Correct genomic integration was confirmed by DNA blotting, as described. Following rapid passage through susceptible BALB/c mice (as described in Depledge *et al*., 2009), these parasites were also maintained in culture as described above.

### Immunoblotting

Whole parasite lysates were separated by SDS-PAGE as described (McKean *et al*., 2001) and blots probed with polyclonal antisera against HASPB (Flinn *et al*., 1994) or SHERP (Knuepfer *et al*., 2001). A monoclonal antibody recognizing the constitutively expressed protein EF1α (clone CBP-KK1, Millipore) or the polyclonal *L. major* anti-*N*-myristoyl transferase (Price *et al*., 2003) were used to control for equivalent protein loading.

For PPG analysis, low-passage promastigotes were grown to late-log-phase and parasite samples collected for lysis and SDS-PAGE as described above. Culture supernatant was fractionated by sequential centrifugation at 2200 *g* (10 min at 3200 rpm) and 100 000 *g* (60 min at 30 000 rpm) for 10 min. The final supernatant was discarded and the pellet lysed in SDS-PAGE gel loading mix prior to SDS-PAGE analysis using a 3 cm, 4% stacking gel and a 12% resolving gel. Blots were probed with anti-LT6 (1:500; the kind gift of Paul Bates, Lancaster University) with detection by ECLplus (Amersham).

### Sand flies and sand fly infections

The colony of *P. papatasi* was maintained at 26°C on 50% sucrose and 14 h light/10 h dark photoperiod as described previously (Benkova and Volf, 2007). Sand fly females were infected by feeding through a chick-skin membrane on heat-inactivated rabbit blood containing 10^6^ promastigotes ml^−1^. Engorged sand flies were maintained in the same conditions as the colony and dissected 2, 5, 9 and 12 days PBM. The location of *Leishmania* infections in the sand fly digestive tract (foregut, SV, thoracic and abdominal midgut, and endoperitrophic and ectoperitrophic space) was determined by dissection and examination by light microscopy. Parasite loads were estimated by two methods: infections seen in the gut *in situ* were graded according to Myskova *et al*. (2008) as light (< 100 parasites per gut), moderate (100–1000 parasites per gut) and heavy (> 1000 parasites per gut). Alternatively, 30–40 guts from females with late infections (10–12 days PBM) were individually dissected into NET 50 and stored in −20°C for qPCR. Sand fly infection experiments were repeated four times for combinations of wild-type (FVI), KO and Kin lines and twice for combinations of FVI, +HASPB and +SHERP lines.

### Quantitative PCR

Extraction of total DNA from dissected sand fly guts was performed using a DNA tissue isolation kit (Roche Diagnostics, Indianapolis, IN) according to the manufacturer's instructions and DNA was eluted in 100 µl of EB buffer. qPCR for detection and quantification of *Leishmania* parasites was performed in Bio-Rad iCycler & iQ Real-Time PCR Systems by using the SYBR Green detection method (iQ SYBR Green Supermix, Bio-Rad, Hercules, CA). The kinetoplast primers described by Mary *et al*. (2004) (forward primer 5′-CTTTTCTGGTCCTCCGGGTAGG-3′ and reverse primer 5′-CCACCCGGCCCTATTTTACACCAA-3′) were used (for more details see Myskova *et al*., 2008). A series of 10-fold dilutions of *Leishmania* promastigote DNA, ranging from 10^4^ to 10^−2^ parasites per reaction, was used to mix with DNA from sand fly females. DNA from uninfected sand flies was used as a negative control.

### Morphometry of parasites

Gut smears of *L. major*-infected females 5, 9 and 12 days PBM were fixed with methanol, stained with Giemsa and examined under the light microscope with an oil-immersion objective. One hundred and sixty randomly selected promastigotes from four sand flies/smears were measured in each combination of *Leishmania* line and time PBM. Body length, flagellar length and body width of parasites were measured and position of the kinetoplast in relation to the nucleus was examined. Four morphological forms were distinguished, based on the criteria of Walters (1993) and Cihakova and Volf (1997): (i) short promastigotes: body length < 14 µm and flagellar length < 2 times body length; (ii) elongated nectomonads: body length ≥ 14 µm; (iii) metacyclic promastigotes: body length < 14 µm and flagellar length ≥ 2 times body length, and (iv) round forms: body width > 4 µm and body length ≤ 7.5 µm include also paramastigotes with kinetoplast lateral to the nucleus. We use here the term short promastigotes derived from the terminology of Walters (1993) (short nectomonad promastigotes) which is the older synonym of leptomonads (leptomonad promastigotes) proposed by Rogers *et al*. (2002). Haptomonads cannot be distinguished in this study as they remain attached to the gut and cannot be measured on gut smears.

### Indirect immunofluorescence

Metacyclic LPG was detected using 3F12 monoclonal antibody from mouse ascites fluid (Sacks and da Silva, 1987). HASPB and SHERP proteins were detected using rabbit polyclonal anti-HASPB and anti-SHERP, both affinity-purified against recombinant protein (Flinn *et al*., 1994; Knuepfer *et al*., 2001).

For cultured parasites, following agglutination with 100 µg ml^−1^ peanut lectin, non-agglutinated cells were fixed in 4% paraformaldehyde and permeabilized in 0.2% Triton X-100 prior to preparation of slides as previously described (Denny *et al*., 2002). Primary antibodies (undiluted 3F12 ascites; anti-HASPB at 1:200 in 1% BSA in PBS) were detected using Alexa Fluor 488- (goat anti-rabbit) or 594- (goat anti-mouse) conjugated secondary antibodies (Invitrogen). Samples were visualized by confocal microscopy using a Zeiss LSM 510 meta with a Plan-Apochromat 63×/1.4 Oil differential interference contrast objective lens and images acquired using LSM 510 version 3.2 software (Carl Zeiss, Jena, Germany).

Gut smears taken from infected sand flies were air-dried on glass slides and fixed with methanol. Non-specific binding was blocked with 1% BSA in PBS (Phosphate Buffered Saline, pH 7.4) for 20 min and the slides then washed and incubated for 30 min with antibodies. 3F12 ascites was used undiluted while anti-HASPB and anti-SHERP antibodies were diluted 1:200 in 1% BSA in PBS. After washing, slides were incubated for 1 h with goat anti-mouse polyvalent FITC-conjugated IgG (Sigma) in dilution 1:250 in 1% BSA in PBS (3F12 antibody assay) or with goat anti-rabbit FITC-conjugated IgG (Sigma) in dilution 1:160 in 1% BSA in PBS (anti-HASPB and anti-SHERP antibodies assays). Slides were then re-washed and after mounting in Vectashield with propidium iodide (Vecta Laboratories) examined under oil-immersion objective in Olympus BX51 fluorescent microscope. For each combination of *Leishmania* line, antibody and time PBM, 100 promastigotes were photographed with an Olympus camera, the images measured with Image J software and classified. These 100 parasites came from at least two different gut smears taken from different sand flies.

### Statistical analysis

Measurements of parasites and the representation of morphological forms were compared using analysis of variance (*post hoc* test) and chi-square test respectively. Results of qPCR were tested with non-parametric Mann–Whitney *U*-test and Kruskal–Wallis test (anova). All the statistical evaluations were performed with statistical software SPSS version16 and Statistica version 6.0.
